# Planktonic food web structure and trophic transfer efficiency along a productivity gradient in the tropical and subtropical Atlantic Ocean

**DOI:** 10.1038/s41598-019-38507-9

**Published:** 2019-02-14

**Authors:** Laia Armengol, Albert Calbet, Gara Franchy, Adriana Rodríguez-Santos, Santiago Hernández-León

**Affiliations:** 1 Instituto de Oceanografía y Cambio Global (IOCAG), Universidad de Las Palmas de Gran Canaria (ULPGC), Unidad Asociada ULPGC-CSIC, Parque Científico Marino de Taliarte, Las Palmas de Gran Canaria, Spain; 2 Institut de Ciències del Mar, CSIC, Passeig Marítim de la Barceloneta 37-49, 08003 Barcelona, Spain

## Abstract

Oligotrophic and productive areas of the ocean differ in plankton community composition and biomass transfer efficiency. Here, we describe the plankton community along a latitudinal transect in the tropical and subtropical Atlantic Ocean. *Prochlorococcus* dominated the autotrophic community at the surface and mixed layer of oligotrophic stations, replaced by phototrophic picoeukaryotes and *Synechococcus* in productive waters. Depth-integrated biomass of microzooplankton was higher than mesozooplankton at oligotrophic stations, showing similar biomasses in productive waters. Dinoflagellates dominated in oligotrophic waters but ciliates dominated upwelling regions. In oligotrophic areas, microzooplankton consumed ca. 80% of the production, but ca. 66% in upwelling zones. Differences in microzooplankton and phytoplankton communities explain microzooplankton diel feeding rhythms: higher grazing rates during daylight in oligotrophic areas and diffuse grazing patterns in productive waters. Oligotrophic areas were more efficient at recycling and using nutrients through phytoplankton, while the energy transfer efficiency from nutrients to mesozooplankton appeared more efficient in productive waters. Our results support the classic paradigm of a shorter food web, and more efficient energy transfer towards upper food web levels in productive regions, but a microbially dominated, and very efficient, food web in oligotrophic regions. Remarkably, both models of food web exist under very high microzooplankton herbivory.

## Introduction

On a global basis, microzooplankton (µZ) graze between 60 and 75% of primary production (PP) daily, whereas mesozooplankton (MZ) consume between 12 and 35%^1^. Therefore, the combined impact of both groups account, on average, for ca. 3/4 of total PP^2^. Given the important role of zooplankton in organic matter turnover, and to fully understand and model the ocean carbon cycle, the rate processes between producers and consumers and their biomass and community structure should be assessed at the ocean basin scale^3^. However, the trophic relationships between consumers and producers are highly variable and difficult to parameterize. For instance, authors have found bottom-up linkage, top-down control, or slight coupling between the different planktonic food web levels in different regions of the ocean^4–7^. This is to be expected, given the complexity and variability observed in systems of different trophic status^8–10^.

Oligotrophic food webs are substantially different to those from productive areas^11–13^. However, general ecological rules should apply irrespective of the ecosystem under investigation (e.g., metabolic theory, Q_10_ concept, etc.). Thus, interconnecting the dynamics of diverse trophic areas is a challenge, and identification of the key processes influencing the dynamics of the marine food web has important implications in understanding the role of these organisms in the fate of carbon in the ocean. Numerous studies have addressed trophic relationships between planktonic organisms in the ocean, however, few studies have covered a wide spectrum of ecological scenarios^14–17^.

The warm and stratified subtropical gyres are oligotrophic areas covering approximately 40% of the planetary surface, and they are expanding 0.8–4.3%·y^−1^ ^18^. Because of the large area they occupy, oligotrophic gyres have an important influence on the contribution of PP and carbon export from the euphotic zone at the global scale^18^. Small cells predominate in these waters, and µZ are more effective than MZ in preying upon phytoplankton, as a result of their similar size to phytoplankton, high growth rates, and high metabolism^19–22^. Growth rates based on chlorophyll *a* (Chla) reported in the literature range from 0.1 to 2 d^−1^ in these systems, probably due to the different phytoplankton responses to nutrient inputs and temperature^23–27^. The major grazers, the µZ, consume up to 70% of the PP in tropical and subtropical systems^28^. Unlike oligotrophic areas where most likely dinoflagellates (Din) are the potential dominant microbial grazers, diatoms (Dia) dominate the autotrophic community in more productive systems^29^. Even in these rich waters, µZ are the major grazers, consuming ca. 60% of the PP^2,30^. Additionally, MZ have been reported as important consumers of µZ in oligotrophic environments and, with less impact, in upwelling systems^31^. Therefore, the relationship between these two important groups of organisms (i.e. µZ and MZ) influences the energy and carbon flow throughout the food web^29^.

In this study we investigated a wide range of different scenarios in the tropical and subtropical regions, from oligotrophic to productive areas. We aimed to understand the trophic relationships from pico- to MZ at the basin scale from 13°S to 25°N in the Atlantic Ocean. Physico-chemical (temperature, salinity, oxygen, and inorganic nutrients) and biological variables (µZ and MZ biomass and µZ grazing) were studied in environments as different as the subtropical gyre and the African upwelling system.

## Results

### Hydrological structure

**Figure 1:**
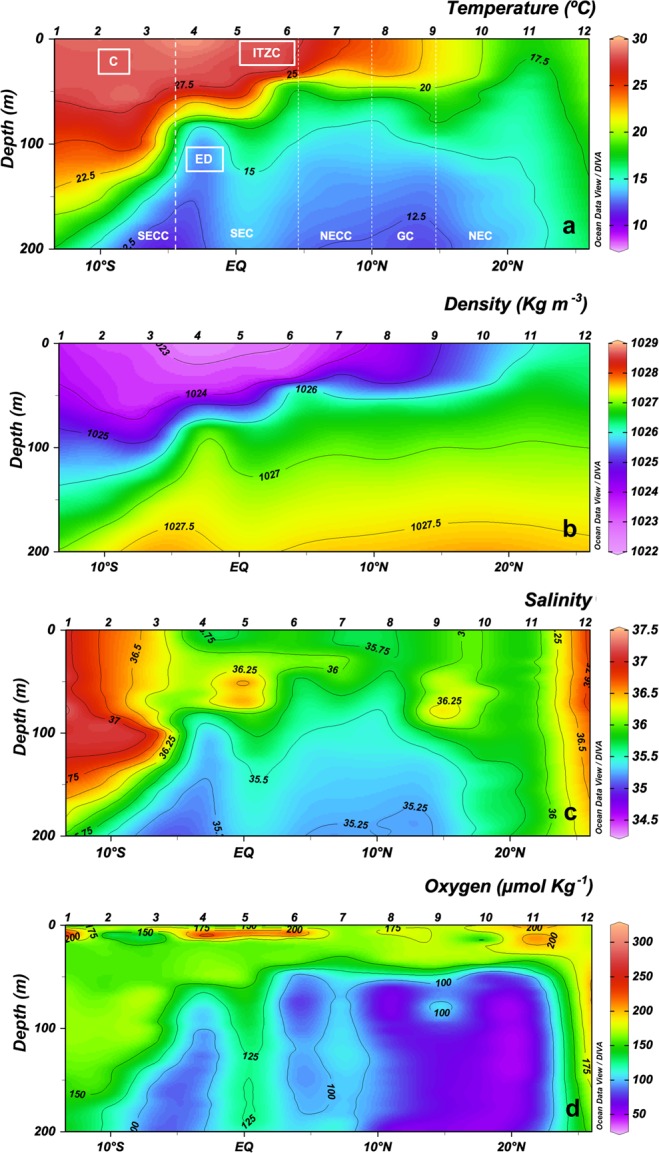
Vertical section (0–200 m) of (**a**) temperature (°C), water currents (South Equatorial Counter Current (SECC), South Equatorial Current (SEC), North Equatorial Counter Current (NECC), Guinea Dome (GD). North Equatorial Current (NEC)) and physical processes (Convergence (C), Equatorial divergence (ED), Intertropical Convergence Zone (ITCZ)); (**b**) density (Kg m^−3^); (**c**) salinity; and (**d**) dissolved oxygen (μmol Kg^−1^) along transect in the Atlantic basin, based on CTD data. Biogeochemical areas are indicated at the top of panels.

We observed a sharp temperature and density gradient along the transect, as expected (Fig. 1). A convergence of the South Equatorial Counter Current (SECC; Reid^32^) showed a deeper thermocline and high salinity (Stations 1 to 3), while the Equatorial Divergence within the South Equatorial Current (SEC) promoted a shallower thermocline and a decrease in dissolved oxygen concentration (Station 4) (Fig. 1). The Intertropical Convergence Zone (ITCZ) showed the slightly deepest thermocline as well as high oxygen concentration levels (between Stations 5 and 6). At Station 8, the North Equatorial Current (NEC) lowered the temperature northward of 10°N and produced an oxygen minimum zone (OMZ). Station 9 showed typical features of the Guinea Dome, characterized by anticlinal thermal and saline structure. The upwelling off Cape Blanc produced cold temperatures and less stratified waters (Stations 10 and 11), while the Canary Current presented waters with high salinity and oxygen concentration (Station 12, Fig. 1).

### Nutrient distribution

**Figure 2:**
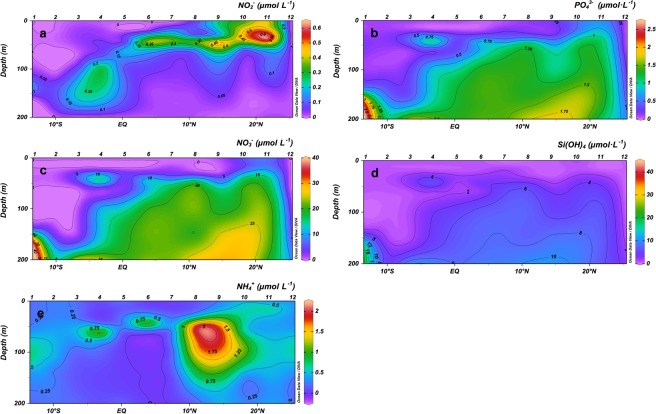
Vertical section (0–200 m) of (**a**) nitrites, (**b**) phosphates, (**c**) nitrates, (**d**) silicates and (**e**) ammonia (μmol L^−1^).

Inorganic nutrient concentrations were higher below the thermocline throughout the transect, as expected (Fig. 2). The highest values for nitrite were found at the Equatorial Divergence, at the mid-ocean upwelling below 50 m depth, at Guinea Dome, and on the surface at the Cape Blanc upwelling (Fig. 2a). Nitrates, phosphates, and silicates showed a core around 50 m depth in the Equatorial Divergence, while from the mid-ocean upwelling (Station 6) to the Cape Blanc upwelling nutrients concentration increased in the mixed layer. The Guinea Dome made an exception because nutrients decreased in the upper layers at this site (Fig. 2b–d). Ammonium levels were slightly higher near the thermocline but showing an important increase (>2 μmol L^−1^) near the Guinea Dome (Stations 8 and 9, Fig. 2e).

### Phytoplankton community

**Figure 3:**
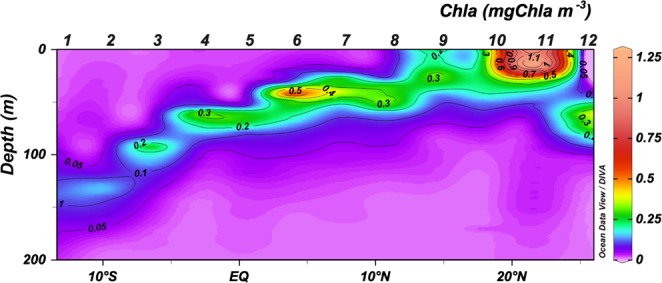
Vertical section (0–200 m) of Chlorophyll *a* (mgChla m^−3^).

**Figure 4:**
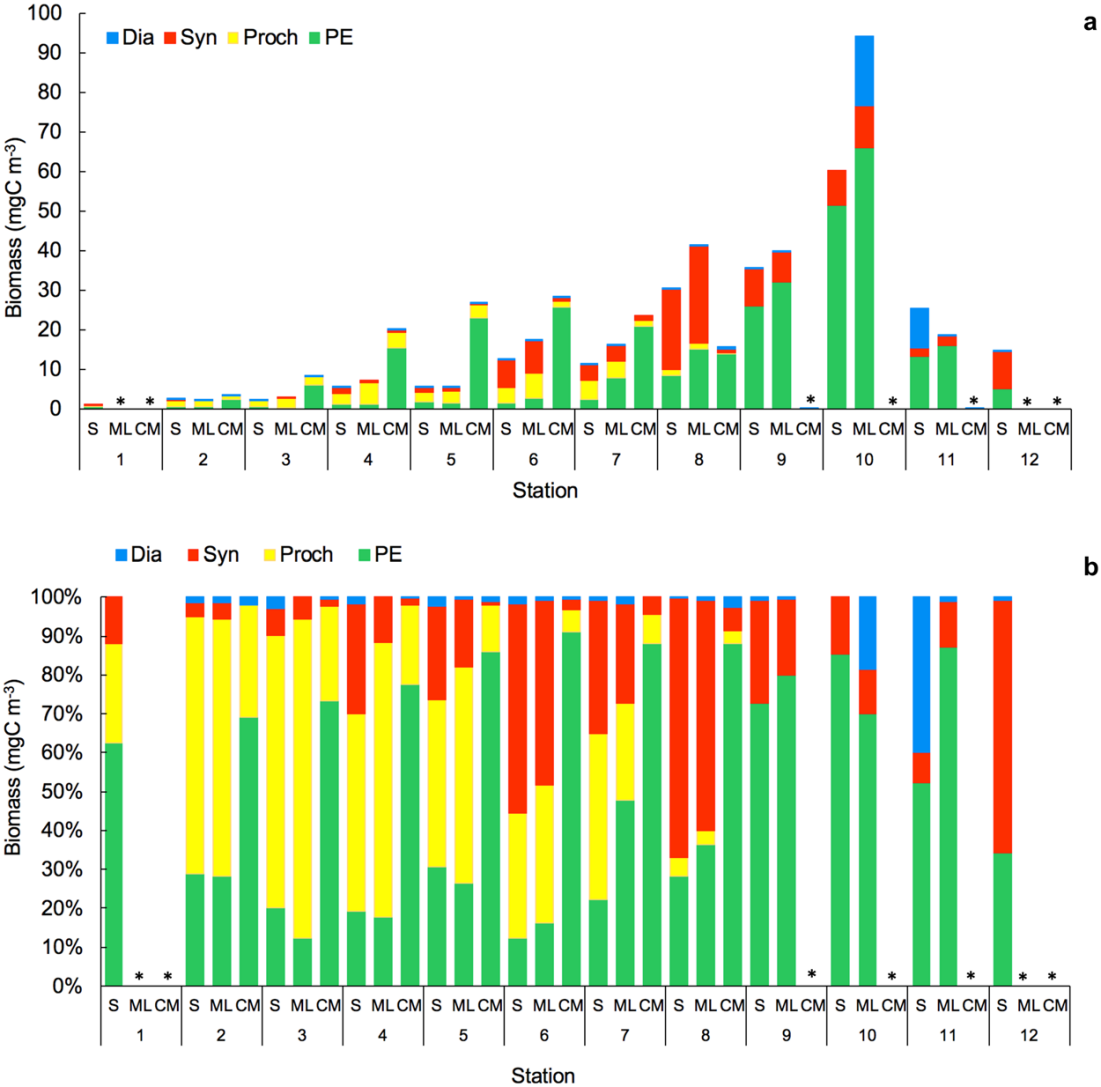
(**a**) Proportion of biomass (%) and (**b**) Biomass (mgC m^−3^) of Cyanobacteria (*Synechococcus*, Syn; *Prochlorochoccus*, Proch; and picoeukaryotes, PE) at the surface layer (5 m depth, S), mixed layer (between 20–30 m depth, ML) and chlorophyll *a* maximum (CM). *No data available.

**Figure 5:**
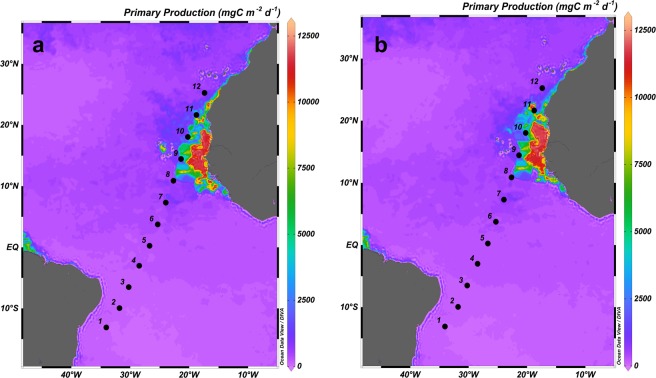
Surface maps of Primary Production (mgC m^−2^ d^−1^) from satellite data during 15–22 April (**a**) and 23–30 April (**b**).

Along the transect, the Chla maximum (CM) followed the base of the thermocline (Fig. 3), which was deeper in the warmest and oligotrophic areas (Stations 1–3), and shallower in the coldest and upwelling-influenced areas (Stations 10 and 11). The CM showed the highest values at the mid-ocean equatorial upwelling and Cape Blanc upwelling. Conversely, the lowest Chla values were observed at the surface and at the mixed layer (ML) in the oligotrophic area (Figs 3 and 4a). The Kendall Rank correlation test showed a positive correlation between Chla and nutrient concentration (τ = 0.69, *p* < 0.001 for NO_3_ + NO_2_; and τ = 0.57, *p* < 0.001 for phosphates). Physical factors, such as temperature and nutrient concentration, as well as MZ biomass explained 85.2% of the variance in the distribution of Chla (PCA and GAM tests, Table 1). The signature of the Guinea Dome and Northwest African upwelling were also conspicuous on the satellite data, showing rather high values of PP (Fig. 5).

**Table 1:** Principal Component Analysis (PCA) and Generalized Additive Model (GAM) for groups of organisms using biological and physical variables as effects; n = 28.

Model	PCA (variance, %)	GAM					
		Residual *Df*	F or t	Deviance explained	R-sq (adj)	GCV	Scale est.
*Chlorophyll a* Terms:	85.2%			71.2%	0.68	75.31	64.55
^+^Temperature			−3.19**				
^+^NO_3_			1.6				
^+^Mesozooplankton			2.3**				
*PE* Terms:	64.1%			99.3%	0.92	39.57	20.93
Temperature		7.65	22.74***				
NO_3_		2.64	9.99**				
Mesozooplankton		8.98	16.8***				
*Synechococcus* Terms:	75.2%			95.8%	0.9	8.05	3.72
NO_3_		2.46	37.64***				
Dinoflagellates		6.24	3.23*				
Mesozooplankton		5.79	19.89***				
*Prochlorococcus* Terms:	81%			81.5%	0.68	1.83	1.00
Temperature		7.52	3.96**				
NO_3_		3.08	1.19				
Microzooplankton		1.00	2.01				
Microzooplankton Terms:	85%			70.6%	0.57	204.4	144.56
Temperature		2.06	9.37**				
Chlorophyll *a*		3.84	4.68**				
Mesozooplankton		1.00	0.003				
Mesozooplankton Terms:	85.2%			85.4%	0.74	53.09	29.45
Temperature		3.02	4.39*				
Chlorophyll *a*		2.50	6.98**				
Microzooplankton		6.74	1.70				

Residual Degrees of Freedom (*Df*).

^+^t-value for linear adjust and F-value for smooth adjust.

Significance level: *p < 0.1; **p < 0.01; ***p < 0.001.

**Figure 6:**
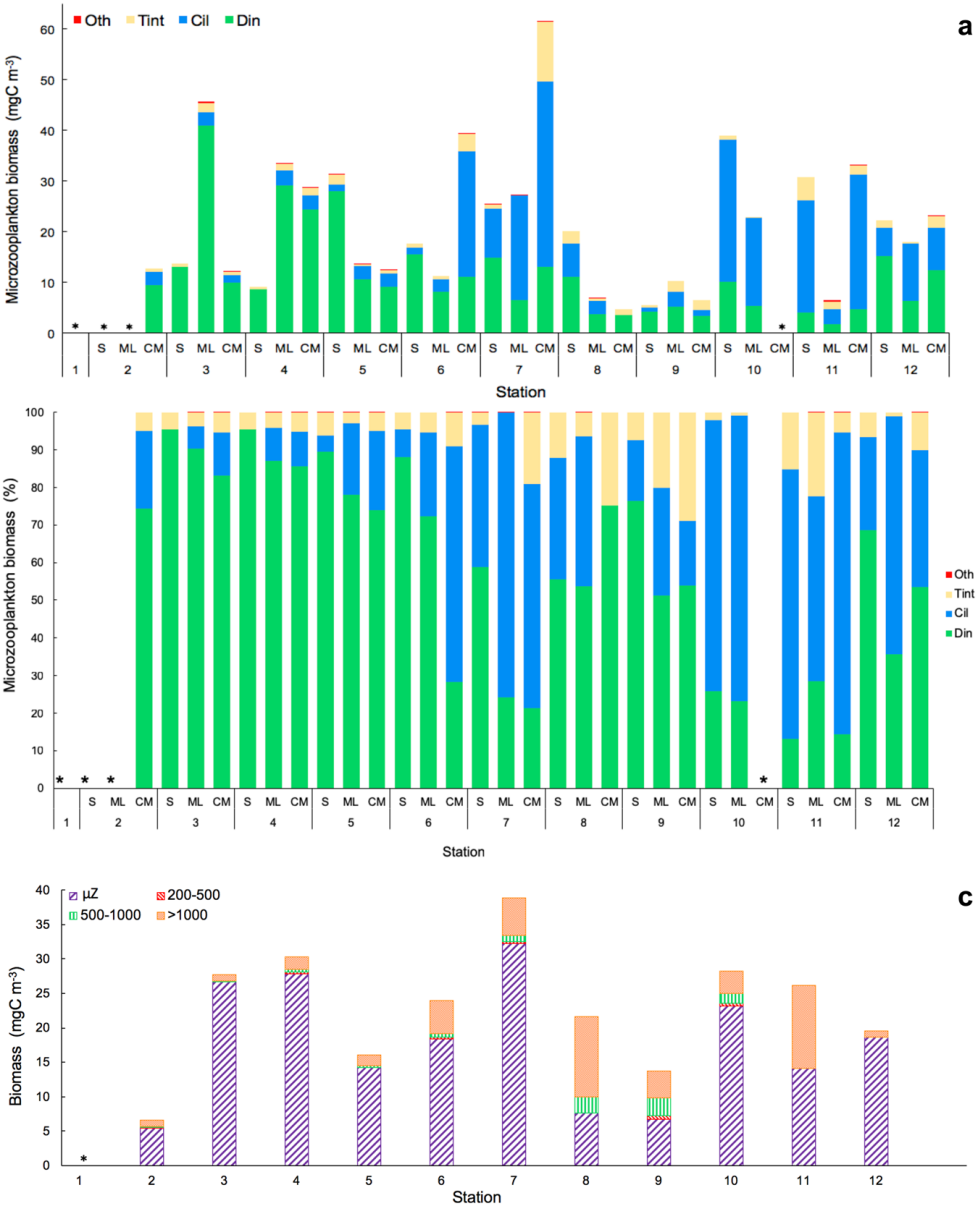
Biomass of dinoflagellates (Din), ciliates (Cil), tintinnids (Tint), and others microzooplankton groups (Oth) (**a**) in mgC m^−3^ and (**b**) in %; (**c**) Integrated biomass (mgC m^−3^) in the water column of microzooplankton (μZ) and different mesozooplankton size-fraction: 200–500 μm (200–500), 500–1000 μm (500–1000) and >1000 μm (>1000). *No data available.

The biomass of phototrophic picoplankton, based on cytometry data, increased from oligotrophic to upwelling regions (Fig. 4), with a further decrease at the Cape Blanc upwelling (Station 11), where Chla showed maximum concentrations (Fig. 3). *Prochlorococcus (Proch)* dominated at the surface and ML at the most oligotrophic and warmest stations and was replaced by *Synechococcus* (*Syn*) in more productive waters. Picoeukaryotes (PE) dominated the autotrophic community at stations with low temperatures and high nutrient availability, such as in the Guinea Dome and Cape Blanc upwelling zone, as well as at the CM throughout all stations (Kendall Rank correlation test τ = 0.61, *p* < 0.001 for NO_3_ + NO_2_) (Fig. 6). 81% of *Proch* and 64.1% of PE biomass variability was explained by temperature, nutrients, µZ, and MZ, whereas *Syn* distribution was determined by temperature (75.2%), µZ, and MZ (PCA and GAM tests, Table 1).

### Micro- and mesozooplankton community

The oligotrophic stations and mid-ocean upwelling showed the highest µZ biomass (mean 20.61 ± 3.49 SE mgC m^−3^), and its importance decreased along the transect (Fig. 6a–c) as temperatures fell. Chla, PE, *Syn*, and MZ explained 85% of µZ biomass variability (PCA and GAM tests, Table 1). Din biomass dominated the microzooplankton in the warmest and stratified waters, comprising 60–80% of total µZ biomass. From the mid-ocean upwelling, Din dominance became irregular with decreasing abundance and an increasing abundance of the naked ciliates (*Cil*) (Fig. 6b). This change in micro-grazer dominance was especially evident in upwelling stations where temperatures decreased sharply. Tintinnids contributed <5% of the total µZ biomass at all stations (Fig. 6b).

MZ biomass increased along the transect (Fig. 6c) with temperature decrease, showing the lowest MZ biomass in the oligotrophic region (mean 4.89 ± 1.64 SE mgC m^−3^) (Fig. 6c). In size terms, the organisms of the MZ with a size >1000 µm dominated the MZ community at all stations, although the biomass of organisms between 500 and 1000 µm increased at Stations 8 and 9.

### Microzooplankton grazing

**Figure 7:**
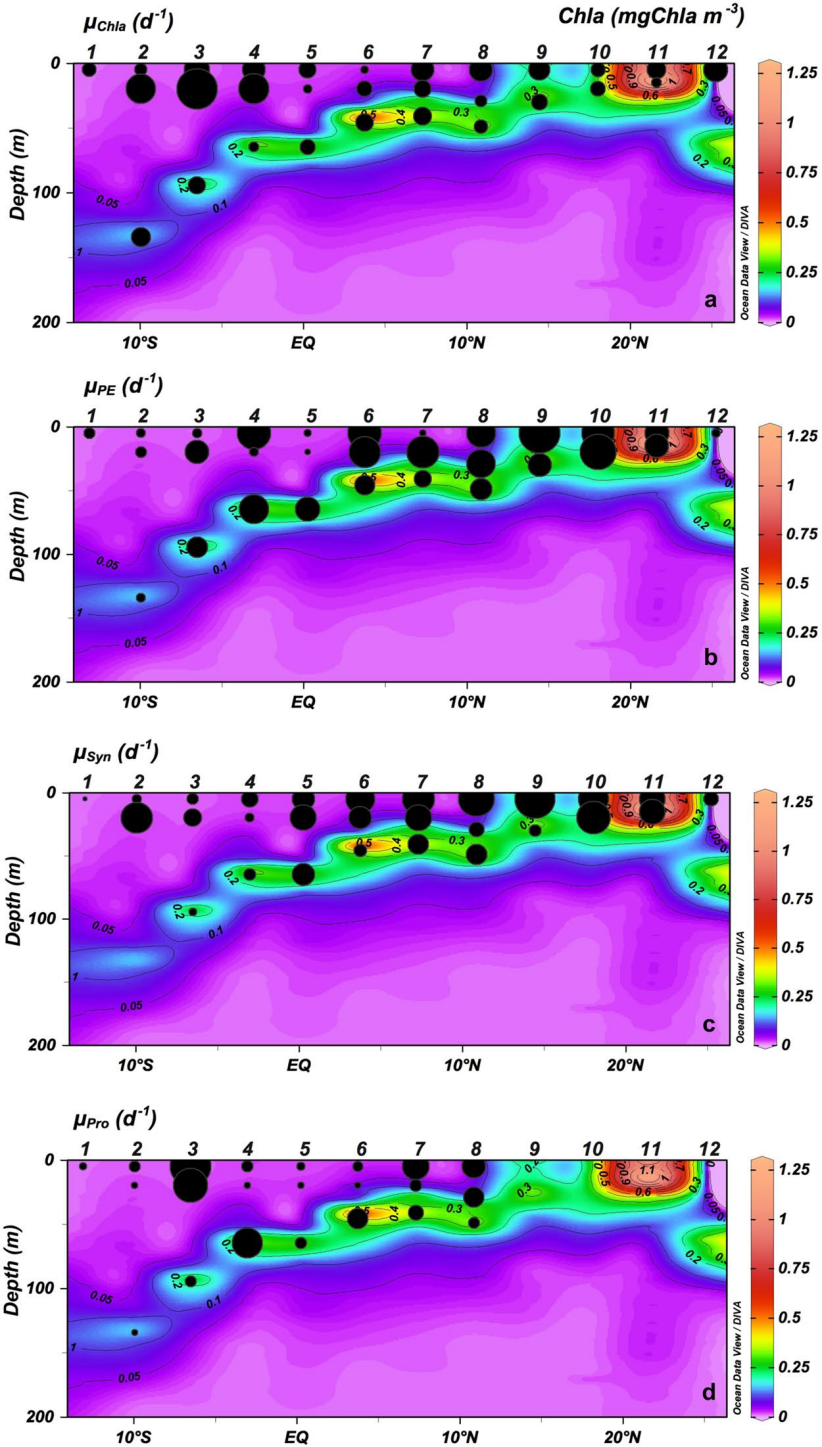
Vertical section (0–200 m) of Chlorophyll *a* (mgChla m^−3^) and potential growth rates (μ, d^−1^) for (**a**) Chlorophyll *a* (μ_Chla_), (**b**) picoeukaryotes (μ_PE_), (**c**) *Synechococcus* (μ_Syn_) and (**d**) *Prochlorococcus* (μ_Pro_).

Potential phytoplankton growth rates based on Chla (µ_Chla_) in the ML were higher at the oligotrophic stations within the SECC and Equatorial Divergence than at other oligotrophic stations (Fig. 7a; Table 2). However, the growth rates of the different groups of autotrophs differed from those based on Chla (Fig. 7, Table 2) showing significant differences between oligotrophic and productive areas (*p* < 0.001; Wilcoxon-Mann-Whitney test). PE and *Syn* potential growth rates (µ_PE_, µ_Syn_) showed slightly higher values at the surface and ML in productive areas (mean 0.52 ± 0.08 SE d^−1^ and 0.65 ± 0.14 SE d^−1^, for PE and *Syn* respectively), and the lowest rates (mean 0.23 ± 0.07 SE d^−1^ for PE and 0.36 ± 0.07 SE d^−1^ for *Syn*) at oligotrophic stations (*p* < 0.001; Wilcoxon-Mann-Whitney test for PE and t-test for *Syn*) (Fig. 7b,c). Potential growth rates for *Proch* (µ_Pro_) were lower than for other picoplankton organisms at all stations except Station 3 (Fig. 7d). At CM, potential growth rates for autotrophic picoplankton and Chla showed non-significant differences between oligotrophic and productive areas (t-test for µ_Chla_ and µ_Syn_; Wilcoxon-Mann-Whitney test for µ_PE_ and µ_Pro_; Fig. 7, Table 2).

**Table 2:** Phytoplankton growth (μ) and microzooplankton grazing (g) rates (d^−1^) for total chlorophyll *a* (Chla), picoeukaryotes (PE), *Synechococcus* (Syn) and *Prochlorococcus* (Proch) from seawater dilution experiments at surface (5 m), mixed layer (20 m) and chlorophyll maximum (CM).

Station	Depth (m)	Growth (d^−1^)				Grazing (d^−1^)			
		μ_Chla_	μ_PE_	μ_Syn_	μ_Proch_	g_Chla_	g_PE_	g_Syn_	g_Proch_
1	5	0.155 ± 0.00	0.073 ± 0.03	0.013 ± 0.01	0.031 ± 0.000	0.177 ± 0.034	0.186 ± 0.014	0.298 ± 0.123	0.114 ± 0.043
2	5	0.119 ± 0.048	0.047 ± 0.027	0.073 ± 0.006	0.071 ± 0.008	0.098 ± 0.005	0.071 ± 0.024	0.265 ± 0.02	0.001 ± 0.024
	20	0.666 ±0.025	0.069 ± 0.03	0.783 ± 0.039	0.001	0.682 ± 0.009	0.086 ± 0.005	0.705 ± 0.027	0.055 ± 0.01
	135 (CM)	0.285 ± 0.041	0.048 ± 0.011	0.001	0.024 ± 0.013	0.179 ± 0.029	0.134 ± 0.008	0	0.047 ± 0.004
3	5	0.502 ± 0.015	0.05 ± 0.031	0.106 ± 0.023	0.952 ± 0.047	0.421 ± 0.013	0.046 ± 0.022	0.18 ± 0.022	0.83 ± 0.031
	20	1.26 ± 0.013	0.318 ± 0.052	0.259 ± 0.059	0.673 ± 0.065	0.769 ± 0.007	0.325 ± 0.006	0.247 ± 0.019	0.718 ± 0.027
	95 (CM)	0.229 ± 0.021	0.252 ± 0.055	0.05 ± 0.017	0.073 ± 0.01	0.167 ± 0.034	0.197 ± 0.019	0.12 ± 0.007	0.057 ± 0.014
4	5	0.400 ± 0.021	0.642 ± 0.104	0.221 ± 0.048	0.074 ± 0.017	0.023 ± 0.051	0.547 ± 0.033	0.249 ± 0.016	0.148 ± 0.022
	20	0.706 ± 0.077	0.045 ± 0.018	0.064 ± 0.005	0.001	0.647 ± 0.021	0.071 ± 0.045	0.081 ± 0.01	0
	65 (CM)	0.077 ± 0.008	0.495 ± 0.05	0.113 ± 0.024	0.527 ± 0.027	0.114 ± 0.027	0.416 ± 0.039	0.092 ± 0.01	0.515 ± 0.01
5	5	0.226 ± 0.048	0.032 ± 0.007	0.396 ± 0.099	0.039 ± 0.023	0	0.058 ± 0.019	0.322 ± 0.041	0.093 ± 0.022
	20	0.061 ± 0.010	0.019 ± 0.009	0.535 ± 0.037	0.001	0.095 ± 0.003	0.062 ± 0.014	0.483 ± 0.012	0.582 ± 0.085
	65 (CM)	0.182 ± 0.018	0.341 ± 0.04	0.397 ± 0.043	0.077 ± 0.012	0.148 ± 0.009	0.303 ± 0.01	0.345 ± 0.027	0.077 ± 0.011
6	5	0.051 ± 0.024	0.642 ± 0.007	0.653 ± 0.067	0.063 ± 0.022	0.081 ± 0.006	0.283 ± 0.009	0.331 ± 0.095	0.093 ± 0.007
	20	0.187 ± 0.024	0.568 ± 0.043	0.408 ± 0.029	0.001	0.161 ± 0.020	0.232 ± 0.027	0.056 ± 0.36	0.544 ± 0.037
	46 (CM)	0.241 ± 0.018	0.225 ± 0.018	0.129 ± 0.008	0.237 ± 0.014	0.205 ± 0.005	0.022 ± 0.023	0.113 ± 0.007	0.249 ± 0.012
7	5	0.408 ± 0.036	0.027 ± 0.014	0.81 ± 0.093	0.409 ± 0.115	0.162 ± 0.015	0	0.183 ± 0.038	0.392 ± 0.011
	20	0.228 ± 0.056	0.593 ± 0.044	0.549 ± 0.025	0.09 ± 0.023	0.153 ± 0.006	0.538 ± 0.014	0.449 ± 0.032	0.593 ± 0.025
	41 (CM)	0.256 ± 0.011	0.169 ± 0.034	0.334 ± 0.049	0.132 ± 0.009	0.181 ± 0.020	0.221 ± 0.015	0.306 ± 0.024	0.178 ± 0.007
8	5	0.426 ± 0.038	0.475 ± 0.079	1.016 ± 0.111	0.24 ± 0.027	0.316 ± 0.017	0.239 ± 0.014	0.658 ± 0.063	0.309 ± 0.011
	29	0.112 ± 0.026	0.47 ± 0.015	0.181 ± 0.047	0.073 ± 0.016	0.144 ± 0.026	0.224 ± 0.075	0.134 ± 0.019	0.493 ± 0.013
	49 (CM)	0.142 ± 0.026	0.265 ± 0.022	0.336 ± 0.029	0.003 ± 0.001	0 ± 0.037	0.294 ± 0.004	0.242 ± 0.025	0.096 ± 0.013
9	5	0.356 ± 0.052	0.939 ± 0.007	1.238 ± 0.037		0.082 ± 0.067	0.621 ± 0.032	0.56 ± 0.022	
	30 (CM)	0.198 ± 0.016	0.313 ± 0.049	0.11 ± 0.031		0.209 ± 0.010	0.29 ± 0.01	0.098 ± 0.012	
10	5	0.159 ± 0.006	0.609 ± 0.023	0.711 ± 0.066		0.040 ± 0.011	0.398 ± 0.036	0.426 ± 0.041	
	20	0.170 ± 0.013	0.737 ± 0.054	0.868 ± 0.064		0.110 ± 0.021	0.68 ± 0.026	0.725 ± 0.011	
11	5	0.273 ± 0.008	0.329 ± 0.047	0.523 ± 0.016		0.250 ± 0.007	0.298 ± 0.044	0.182 ± 0.021	
	15 (CM)	0.076 ± 0.026	0.315 ± 0.047	0.516 ± 0.013		0	0.311 ± 0.005	0.497 ± 0.01	
12	5	0.442 ± 0.080	0.043 ± 0.009	0.173 ± 0.025		0	0.079 ± 0.019	0.115 ± 0.029	

Negative growth and grazing rates were converted to 0.001 and 0, respectively. Note *Proch* were not present at stations 9 to 12. Values (mean ± SE).

**Figure 8:**
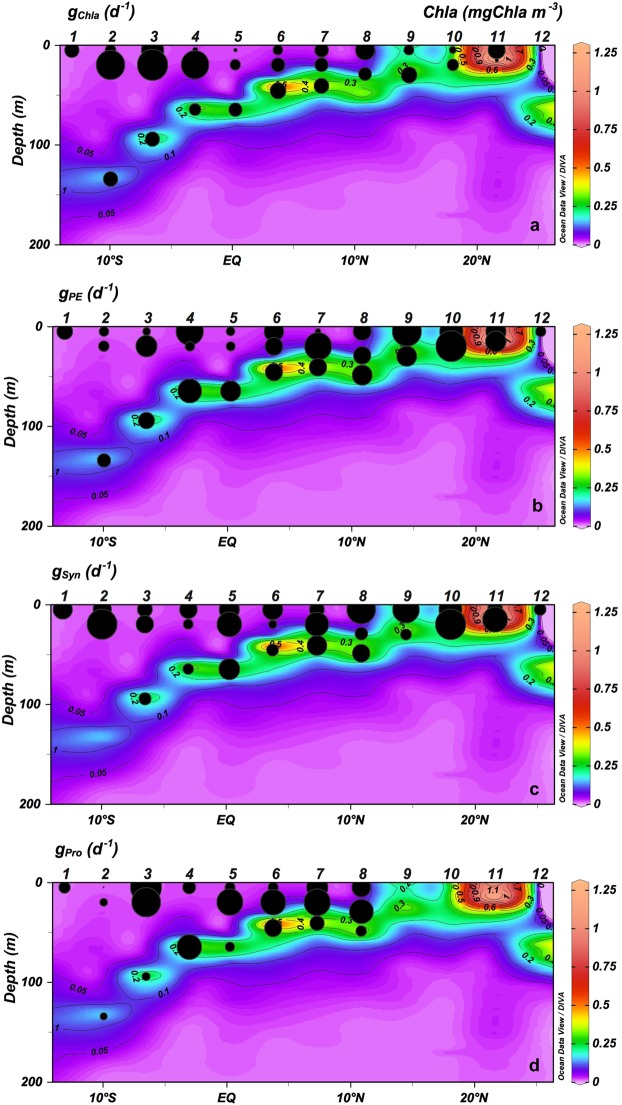
Vertical section (0–200 m) of Chlorophyll *a* (mgChla m^−3^) and microzooplankton grazing rates (g, d^−1^) for (**a**) Chlorophyll *a* (g_Chla_), (**b**) picoeukaryotes (g_PE_), (**c**) *Synechococcus* (g_Syn_) and (**d**) *Prochloroccocus* (g_Pro_).

**Figure 9:**
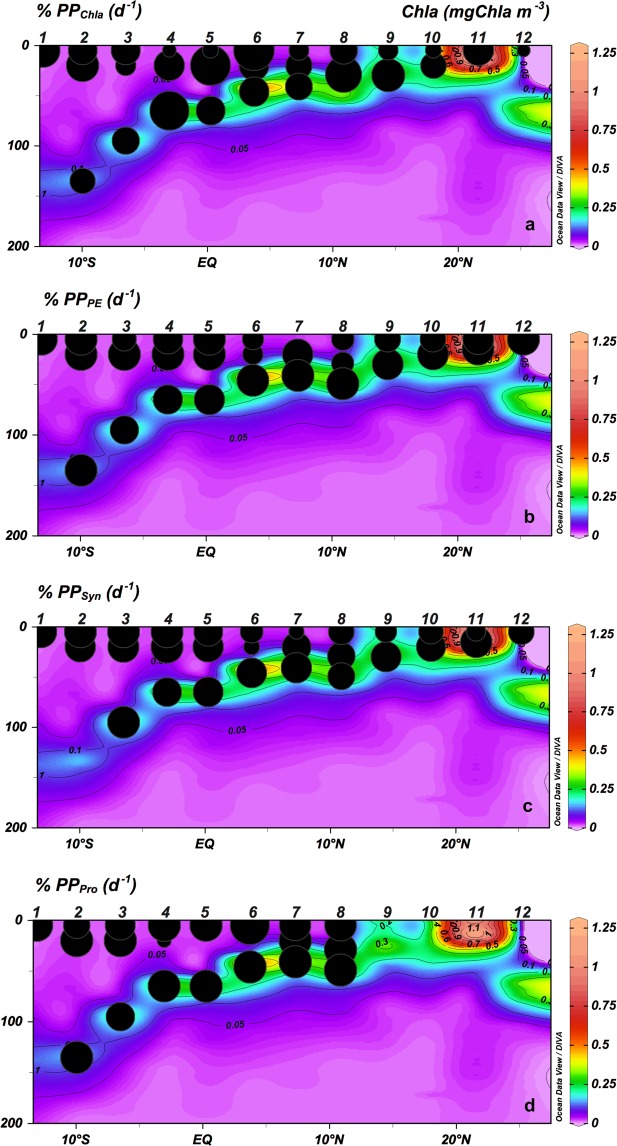
Vertical section (0–200 m) of Chlorophyll *a* (mgChla m^−3^) and microzooplankton grazing on potential phytoplankton production (% PP) for (**a**) Chlorophyll *a* (% PP_Chla_), (**b**) picoeukaryotes (% PP_PE_), (**c**) *Synechococcus* (% PP_Syn_) and (**d**) *Prochlorococcus* (% PP_Pro_).

At the surface and ML, µZ grazing rates on phytoplankton based on Chla (g_Chla_) showed the highest rates at SECC and Equatorial Divergence (Stations from 1 to 4) (Fig. 8A; Table 2). Also, at the surface and ML, grazing rates on PE (g_PE_) and *Syn* (g_Syn_) were significantly lower at oligotrophic stations (0.19 ± 0.05 SE for PE and 0.28 ± 0.05 SE for *Syn*) than at productive stations (0.38 ± 0.06 SE for PE and 0.41 ± 0.09 SE for *Syn*) (*p* < 0.001 Wilcoxon-Mann-Whitney for PE; and p < 0.01 t-test for *Syn*) (Fig. 9b,c; Table 2). The µZ grazing rates on *Proch* (g_Proch_) were higher at the surface and ML at stations with a shallower thermocline (Stations 5 to 8; Fig. 8d; Table 2). At CM, µZ grazing rates of Chla, PE, *Syn* and *Proch* showed a non-significant difference between oligotrophic and productive regions (Wilcoxon-Mann-Whitney test for Chla, *Syn* and *Proch*; t-test for PE) (Fig. 8, Table 2). Overall, µZ grazing rates for all organisms were lower at the CM than in the upper layers (Fig. 8, Table 2).

The ratio of grazing rates to phytoplankton growth (g/μ) provided an estimate of the proportion of the potential PP consumed by microbial grazers (%PP). Based on Chla the %PP_Chla_ showed non-significant differences (t-test) from oligotrophic to upwelling areas at the surface and ML (Fig. 9a). In the same water column range, the impact upon PE (%PP_PE_) and *Syn* (%PP_Syn_) were higher in the oligotrophic areas (134.75 ± 25.03 SE for PE; 108.01 ± 19.04 SE for *Syn*) than in the upwellings (79.15 ± 7.28 SE for PE; 69.01 ± 8.63 SE for *Syn*) (*p* < 0.05 for PE and *p* < 0.01 for *Syn* Wilcoxon-Mann-Whitney test), while the impact of grazers on *Proch* (%PP_Proch_) increased at the surface with more shallow thermoclines, except in the equatorial regions (Wilcoxon-Mann-Whitney test) (Fig. 9b–d).

### Diel growth and grazing rates

**Figure 10:**
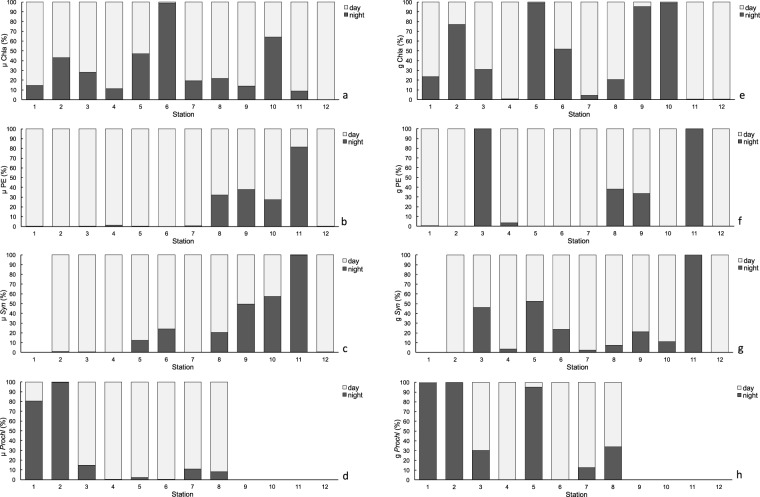
Proportion of phytoplankton potential growth (μ) rates for (**a**) Chlorophyll *a* (Chla), (**b**) picoeukaryotes (PE), (**c**) *Synechococcus* (Syn) and (**d**) *Prochlorococcus* (Proch) and microzooplankton grazing (**g**) rates for (**e**) Chlorophyll *a* (Chla), (**f**) picoeukaryotes (PE), (**g**) *Synechococcus* (Syn) and (**h**) *Prochlorococcus* (Proch) during day (light bars) and night (dark bars) hours at each station.

No clear pattern of diel growth and grazing were observed based on total Chla (Fig. 10a). However, a more detailed study of different groups of plankton showed different daily patterns. PE and *Syn* displayed a clear rhythm in both growth and grazing, with higher rates during the day, while this pattern vanished in upwelling waters (Fig. 10b,c). *Proch* showed higher growth and grazing rates during night in the most oligotrophic and stratified areas (Stations 1 and 2), but the rhythm was the opposite in the Equatorial Divergence (Stations 4 and 5, Fig. 10d, Table 3).

**Table 3:** Phytoplankton growth (μ) and microzooplankton grazing (g) rates (d^−1^) for total chlorophyll *a* (Chla), picoeukaryotes (PE), *Synechococcus* (Syn) and *Prochlorococcus* (Proch) from superficial waters dilution experiments (5 m) during daylight and night hours.

Station	Time	Growth (h^−1^)				Grazing (h^−1^)			
		μ_Chla_	μ_PE_	μ_Syn_	μ_Proch_	g_Chla_	g_PE_	g_Syn_	g_Proch_
1	Day	n.s	0.037 ± 0.004		0.002 ± 0.002	n.s	0.23 ± 0.001		0.000 ± 0.001
	Night	n.s	0.001		0.010 ± 0.011	n.s	0.001		0.015 ± 0.002
2	Day	0.006 ± 0.005	0.049 ± 0.021	0.010 ± 0.001	0.001	0.002 ± 0.002	0.074 ± 0.007	0.033 ± 0.004	0.000 ± 0.014
	Night	0.004 ± 0.001	0.001	0.001	0.024 ± 0.011	0.006 ± 0.002	0.001	0.001	0.023 ± 0.006
3	Day	0.029 ± 0.000	0.028 ± 0.005	0.017 ± 0.001	0.059 ± 0.003	0.015 ± 0.001	0.001	0.006 ± 0.002	0.037 ± 0.007
	Night	0.011 ± 0.000	0.001	0.001	0.010 ± 0.002	0.007 ± 0.000	0.005 ± 0.004	0.005 ± 0.001	0.016 ± 0.002
4	Day	0.030 ± 0.003	0.052 ± 0.014	0.031 ± 0.001	0.022 ± 0.006	0.013 ± 0.018	0.043 ± 0.004	0.019 ± 0.003	0.016 ± 0.003
	Night	0.004 ± 0.001	0.001	0.001	0.001	0	0.002 ± 0.001	0.001 ± 0.001	0.001
5	Day	0.010 ± 0.003	0.029 ± 0.007	0.025 ± 0.009	0.005 ± 0.001	0	0.026 ± 0.001	0.008 ± 0.004	0.000 ± 0.002
	Night	0.009 ± 0.005	0.001	0.003 ± 0.001	0.001	0.014 ± 0.004	0.001	0.009 ± 0.001	0.004 ± 0.002
6	Day	0.001	0.056 ± 0.001	0.032 ± 0.007	0.038 ± 0.004	0.003 ± 0.003	0.087 ± 0.001	0.017 ± 0.001	0.030 ± 0.003
	Night	0.010 ± 0.000	0.001	0.010 ± 0.000	0.001	0.004 ± 0.002	0.001	0.005 ± 0.004	0.001
7	Day	0.027 ± 0.001	0.017 ± 0.008	0.076 ± 0.003	0.026 ± 0.005	0.013 ± 0.002	0.013 ± 0.001	0.014 ± 0.003	0.024 ± 0.005
	Night	0.007 ± 0.002	0.001	0.001	0.003 ± ± 0.001	0.001 ± 0.002	0.001	0.000 ± 0.004	0.003 ± 0.003
8	Day	0.028 ± 0.004	0.019 ± 0.007	0.053 ± 0.008	0.021 ± 0.007	0.021 ± 0.003	0.008 ± 0.002	0.044 ± 0.002	0.012 ± 0.000
	Night	0.008 ± 0.001	0.009 ± 0.004	0.014 ± 0.002	0.002 ± 0.003	0.005 ± 0.001	0.005 ± 0.001	0.004 ± 0.002	0.006 ± 0.000
9	Day	0.026 ± 0.005	0.034 ± 0.002	0.034 ± 0.007		0	0.025 ± 0.001	0.030 ± 0.001	
	Night	0.004 ± 0.000	0.021 ± 0.002	0.034 ± 0.003		0.002 ± 0.000	0.013 ± 0.001	0.008 ± 0.001	
10	Day	0.005 ± 0.001	0.028 ± 0.004	0.016 ± 0.005		0	0.046 ± 0.001	0.027 ± 0.003	
	Night	0.009 ± 0.001	0.011 ± 0.002	0.021 ± 0.001		0.015 ± 0.002	0.001	0.003 ± 0.002	
11	Day	0.021 ± 0.001	0.003 ± 0.002	0.001		0.019 ± 0.001	0.001	0.001	
	Night	0.002 ± 0.000	0.012 ± 0.003	0.026 ± 0.001		0	0.014 ± 0.001	0.015 ± 0.001	
12	Day	0.047 ± 0.004	0.021 ± 0.008	0.017 ± 0.003		0.018 ± 0.002	0.013 ± 0.003	0.019 ± 0.001	
	Night	0.0001	0.001	0.001		0	0.001	0.001	

Negative growth and grazing rates were converted to 0.001 and 0 respectively. Note *Proch* were not present from station 9 to 12. Values (mean ± SE).

### Trophic transfer efficiency

The ratio between the biomass of upper and lower trophic levels can be used as a proxy measure of the trophic transfer efficiency within the food web. Thus the ratio of Chla:(NO_2_ + NO_3_) showed that each µM of N sustained, on average, 22.9 μg C of phytoplankton (±16.86 SE) in oligotrophic regions, and 2.6 μg C of phytoplankton (±0.74 SE) in productive regions. The ratio between biomass of µZ:(NO_2_ + NO_3_) showed that each µmol of N supported 27.9 μg C of µZ (±12.98 SE) in oligotrophic zones, whereas for productive areas this decreased to 2.9 (±0.68 SE) µg C of µZ. For MZ, the ratio between their biomass and NO_2_ + NO_3_ resulted in lower values than for µZ at oligotrophic stations (mean 5.8 μg of MZ ± 1.26 SE), while at productive stations values were higher than for µZ (mean 14.2 μgC of MZ ± 7.83 SE). The carbon transferred from phytoplankton to µZ (µZ biomass:phytoplankton biomass) averaged 3.9 ± 0.68 SE at oligotrophic stations, and decreased to 0.70 ± 0.39 SE at productive stations. Using the same quotient for MZ, in oligotrophic areas the ratios were slightly lower (mean 0.92 ± 0.44 SE) than in productive areas (mean 1.28 ± 0.33 SE). MZ biomass supported by µZ biomass averaged 0.15 ± 0.03 SE in oligotrophic areas, and 1.44 ± 0.33 SE in the upwelling region.

## Discussion

### Major differences between oligotrophic and productive zones: from organismal abundances to trophic transfer efficiencies

The main finding of this study was the close relationship between the distribution and trophic relationships of the planktonic organisms with the physical variables characterizing each geographical region. However, for the sake of simplicity and in spite of the distinctive characteristics of the areas surveyed, we will focus in this section on the oligotrophic and more productive zones, merging the different regions studied into these two categories. In this regard, at the very base of the marine food web, we found that prokaryotes dominated the autotrophic community in oligotrophic areas^33^, most likely because they are more efficient than protists at assimilating nutrients at low concentrations due to their higher cell surface-to-volume ratio^34^. In particular, *Proch* was more abundant in the oligotrophic and warmest waters, whereas *Syn* and PE showed higher biomass in colder and nutrient richer waters (Fig. 4). Differences in their cell structure and physiology may explain this zonation, already reported in other studies^35–37^. *Proch* and *Syn* differ in size and light-harvesting antenna systems and the former is unable to use nitrates, whereas *Syn* uses them as a main source of N (for a review, see^38–41^). Moreover, *Proch* takes up phosphate in nutrient-depleted zones as a result of phosphate-specific acquisition genes, which gives these organisms an advantage in oligotrophic areas^42,43^. These features explained their dominance at the surface and ML in the South Atlantic gyre and Equatorial Divergence. Higher PE biomass occurred in areas with relatively high concentration of nutrients, as in the CM and upwelling regions, in accordance with observations by Tarran^44^. Also, as expected, Dia made a large contribution to the biomass at the upwelling station although they did not dominate the community, as also observed by Marañón^45^. The strong relationship between NO_2_ + NO_3_ and primary producers is mainly because NO_3_ is the most commonly consumed and reduced form of nitrogen for building organic molecules^46^ and NO_2_ plays an intermediary role in the global cycles of nitrogen and carbon and in microbial metabolism. The biotic responses to nutrient concentration can be direct, such as shifts in phytoplankton community composition, or indirect, such as shifts in grazer community composition^47,48^. It is well known, however, that the biomass and distribution of phytoplankton do not solely depend on nutrient availability or temperature; grazing is also an important factor shaping autotrophic communities^49,50^. Our results, similar to those of Calbet and Landry^30^, show that at the surface and ML of the oligotrophic ocean µZ consumed approximately 78% of the PP, whereas in upwelling areas consumption was slightly lower (~66%, Fig. 9). The µZ of oligotrophic regions showed low efficiency in consuming *Proch* (Figs 9d and 10d)^51,52^; however, µZ grazing rates on *Proch* rose at the ITCZ and mid-ocean upwelling stations, coinciding with an increase in PE (Fig. 6b), which have been documented to be efficient mixotrophs^43,53,54^. Therefore, high grazing rates on *Proch* in those areas may be due to a cascade effect where MZ (which increased their biomass) consume µZ (decreasing their biomass) (Fig. 6a), releasing PE from grazing pressure and increasing their biomass, which in turn increases *Proch* consumption (τ = −0.27, *p* < 0.05; Kendall Rank correlation test between biomass of *Proch* and PE). *Syn* consumption was similar throughout the basin, indicating that Din, which dominated the µZ community in oligotrophic regions (Fig. 6c), and *Cil* consume them at similar rates (e.g. refs^55–57^). In warm oligotrophic regions, where prey are smaller and less numerous, Din dominated the microplankton community (Fig. 6c), as opposed to upwelling areas, were the µZ was dominated by *Cil*. It is known that copepods show a low preference for predation upon Din^58,59^, and show preference for *Cil* (e.g.^60^). This copepod prey selectivity could explain the decrease in µZ biomass compared to MZ in the upwelling areas. Furthermore, higher predation levels on µZ released PE and *Syn* from grazing pressure, facilitating a rise in their biomass (e.g.^60^). A fingerprint of this cascade effect was the positive correlation between MZ biomass and picoautotroph cells.

Overall, in the oligotrophic Atlantic, each µM of N supported far more phytoplankton than in the Northwest African upwelling. This result is not surprising because oligotrophic food webs are known to recycle nutrients more efficiently, also allowing for a proportionally higher biomass of µZ than very productive ones^61–63^. The proportional increase in µZ biomass at the oligotrophic stations did not imply an increase in biomass transfer upwards to MZ, since in oligotrophic environments the carbon of µZ that supported MZ is smaller than in the upwelling areas. These results demonstrate the bottom-up control of µZ in oligotrophic areas, and suggest a closer link between MZ and µZ in upwelling regions. Moreover, each µM of N supports more MZ at productive sites, manifesting the higher linear transfer efficiency of energy in productive ecosystems than in oligotrophic ecosystems^62,63^. Therefore, our results regarding food web trophic efficiency back up the paradigm of much more efficient recycling in oligotrophic conditions, but with an overall lower linear energy transfer towards higher trophic levels. In other words, our data fully support the existence of a strong microbial loop in oligotrophic areas and a more classic food chain in more productive regions (e.g. refs^64–66^). Interestingly, these findings do not contradict the fact that µZ grazing can be very high in productive regions as well, as occurred here, and actually merge the settled paradigms of food web structure (microbial loop for oligotrophic areas and classic food chain for upwellings) with the predominance of the µZ grazing over the MZ in marine ecosystems^2,30^.

### Vertical zonation

The CM in oligotrophic areas is formed as a result of the photoacclimation of the cells and/or an increase in phytoplankton growth due to nutrient diffusion through the thermocline. The biomass-specific grazing on Chla and upon each autotrophic group was lower in the CM than at the surface and ML (Fig. 8). As hypothesized by Landry *et al*.^67^, low grazing rates in areas with high availability of resource, as in the CM, could be a consequence of low concentrations of µZ. Moreover, previous studies found lower growth rates in this environment than in the ML, suggesting a low turnover of the phytoplankton community^25,68,69^, or as in our case, may be the result of an overestimation of phytoplankton production due to our assessment of the potential growth of autotrophic organisms. Worden and Binder^70^ found non-significant differences between growth rates with and without nutrient addition treatments in oligotrophic areas, indicating that growth rates respond to nutrient enrichment at time scales greater than 24 hours or that there may be a lack of nutrient limitation due to fast recycling. If this were the case in our study we should consider the estimated potential growth rates at oligotrophic stations similar to the real rates. Conversely, growth rates based on Chla (Fig. 7a) in surface layers at the productive stations (the mid-ocean upwelling, Guinea Dome and Northwest African upwelling) were similar to those obtained by Marañón *et al*.^25^ where, as in this study, the nitracline occurred at a similar depth.

### Diel cycles of microzooplankton grazing

Previous studies showed that daily variations in phytoplankton in oligotrophic areas were more important than seasonal or annual changes^71–73^. Certainly cloud cover, sinking, advection, and turbulence transporting cells between darkness and full sunlight^74^ modify the intensity of light experienced by cells in the ocean and may have important consequences on phytoplankton growth. In general, light controls cell cycles in many phytoplankters either directly or by adjusting the biological clock^75,76^. For picoplankton, cell division begins near dusk, with *Syn* starting the process, followed by *Proch*, and finally PE^77,78^. Conversely, cell-biomass increases during daylight hours^78–80^, as we observed at the oligotrophic stations (Fig. 10).

Diel cycles of growth have also been identified for µZ species, such as *Gymnodinium* sp.^81^ or *Coxiella* sp.^82^, which showed higher growth rates during daylight, with a few exceptions^83^. Likewise, specific protozoan grazing activity seems to occur mostly during the day^82,84–87^. The reasons for this rhythm could be endogenous circadian cycles, light-aided digestion, or diel variations in phytoplankton stoichiometry^82,83,85–87^. Recently, Arias *et al*.^85^ have suggested that the diel rhythms in µZ were inverse to those of their consumers in order to avoid being more conspicuous during grazing and, therefore, being more prone to predation (i.e. copepods^14,88,89^). Arias *et al*.^85^ also found that diel rhythms of feeding were modulated by hunger and satiation; only satiated protozoans showed full amplitude diel feeding rhythms. A similar response to food availability was also observed in copepods^14^. However, contrary to expectations, diel feeding rhythms in upwelling areas were fuzzy compared to areas with low food availability. We propose two alternative hypotheses to explain this. On one hand, species adapted to low food environments may have satiation thresholds at lower concentrations than those adapted to richer environments. On the other hand, given the specificity of the diel feeding response^84,85^, it is possible to explain the variations in diel feeding behaviour by changes in the composition of the µZ community. Backing up this hypothesis, we found oligotrophic areas being dominated by Din (usually showing more evident diel grazing rhythms than *Cil*^84^), whereas the µZ of more productive waters, mostly dominated by *Cil*, seems to be highly species-specific in their diel behaviours^84^.

### Summary

In summary, across the tropical and subtropical Atlantic Ocean, we found a close relationship between physico-chemical variables and the distribution of planktonic organisms. These changes in distribution and species composition in turn drive the trophic relationships within plankton, consolidating the paradigms of a more complex and efficient nutrient recycling microbial food web in the oligotrophic ocean compared with a “classic” and shorter one in more productive areas.

## Material and Methods

### Sampling and hydrographic measurements

**Figure 11:**
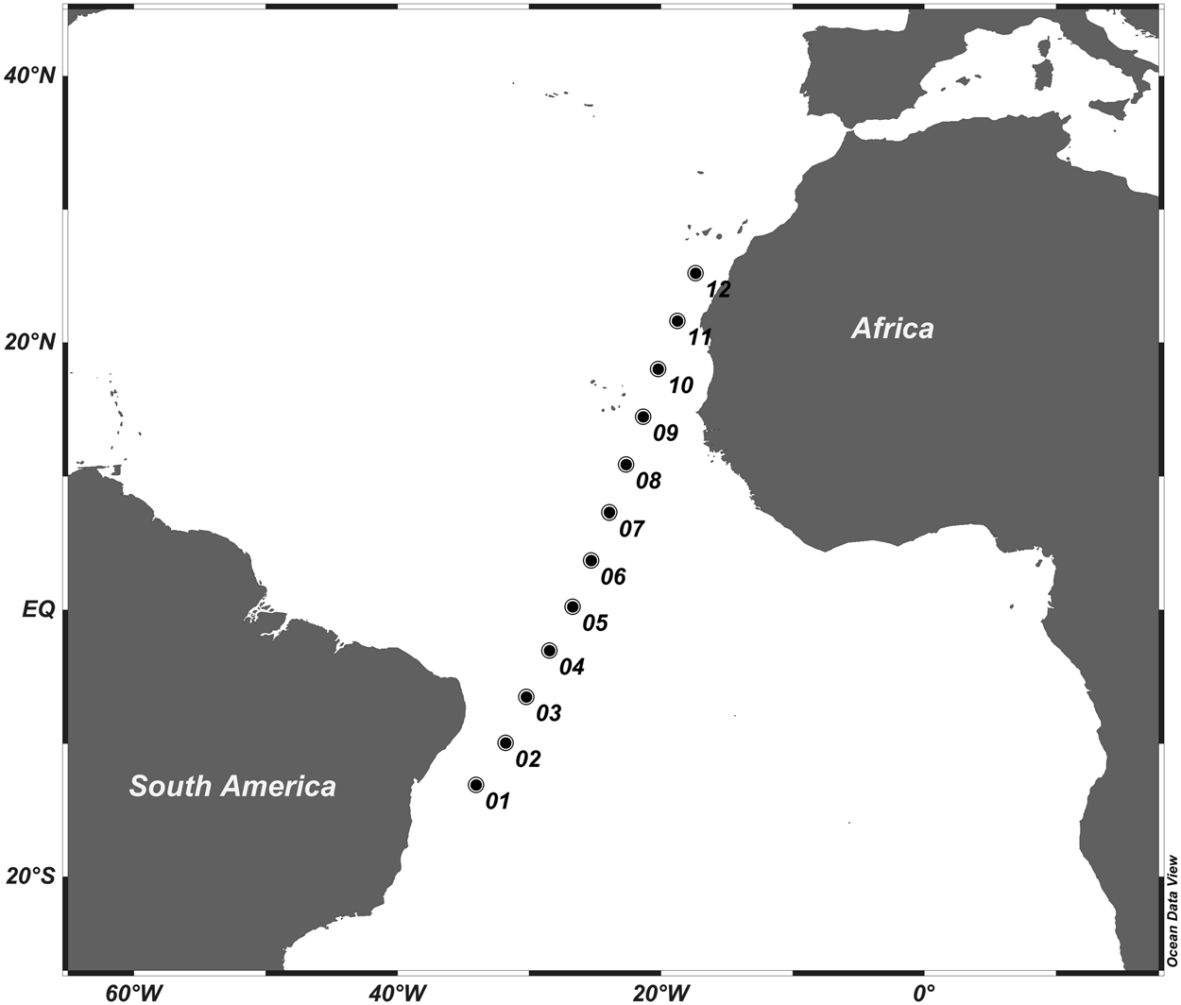
Map of the study area across the Atlantic Ocean.

Sampling took place from 5^th^ to 29^th^ April, 2015 on board the R.V. *Hespérides* from Salvador da Bahia (Brazil) to Canary Islands (Spain). Twelve stations were sampled between 13°S-25°N (Fig. 11, Table 4), and at each station two casts were conducted using a General Oceanics rosette equipped with 24 L PVC Niskin bottles and Seabird 911-plus CTD equipped with a Seapoint Chlorophyll Fluorometer and a Seabird-43 Dissolved Oxygen Sensor. The first cast was carried out down to 3500 m depth during night, and the second cast was carried out from the surface to 200 m depth during daylight hours. Vertical distribution of the photosynthetically active irradiance (PAR, 400–700 nm) was measured using a radiometer Biospherical/Licor installed in the rosette sampler. Water samples to calibrate dissolved oxygen sensor were collected with Niskin bottles along all the water column.

**Table 4:** Location of the studied stations and initial conditions for microzooplankton grazing experiments.

Station	Latitude	Longitude	Depth (m)	Temperature (° C)	Salinity	Dissolved O_2_ (µmol Kg^−1^)
1	−13.12	−34.05	5	28.54	37.02	236.17
2	−9.96	−31.79	5	28.79	36.66	222.73
			20	28.43	36.66	156.31
			135	21.74	36.65	157.71
3	−6.51	−30.22	5	28.74	36.35	184.32
			20	28.6	36.35	155.02
			95	23.95	36.46	177.18
4	−3.03	−28.46	5	29.39	35.75	314.15
			20	28.65	36.01	159.68
			65	22.24	36.20	123.94
5	0.25	−26.70	5	28.45	35.78	255.37
			20	28.10	35.96	159.48
			65	22.64	36.42	146.71
6	3.73	−25.32	5	28.14	35.89	252.51
			20	27.86	35.91	158.43
			46	18.65	35.78	112.55
7	7.30	−23.93	5	25.73	35.73	199.51
			20	25.04	35.75	166.03
			41	21.26	36.00	145.73
8	10.87	−22.65	5	24.13	35.73	210.41
			29	23.72	35.76	169.47
			49	21.18	35.72	160.18
9	14.44	−21.36	5	22.09	35.90	173.51
			30	21.92	35.90	172.38
10	18.04	−20.22	5	20.10	36.00	177.00
			20	20.07	35.99	173.88
11	21.63	−18.76	5	17.98	35.91	163.43
			15	17.69	35.90	205.50
12	25.24	−17.38	5	19.32	36.64	181.81

### Nutrients and oxygen

Inorganic nutrients were sampled from hydrographic bottles with polyethylene tubes and stored frozen (−20 °C) until their analysis in the laboratory. Samples were analysed with a QuAAtro 39-SEAL Analytical AutoAnalyzer following the protocol by Armstrong *et al*.^16^. On board oxygen calibration was carried out with the potentiometric end-point Winkler method^90^.

### Chlorophyll a and picoplankton

Chla samples were taken at 5 levels from the surface to 200 m depth in order to calibrate the fluorescence sensor installed in the rosette. Samples of 500 mL were collected from the Niskin bottles, filtered through 25 mm Whatman GF/F filters and stored frozen until their analysis. In the laboratory, pigments were extracted in cold acetone (90%) for 24 h and analysed using an AU TurnerDesigns bench fluorometer previously calibrated with pure Chla (Sigma Aldrich) according to Yentsch & Menzel^91^ and acidified following Welschmeyer^92^. Chla concentration was converted to carbon assuming a C:Chl of 50^93^ since conversion ratio for the studied area ranged from 30 to 80, being more quoted around 50.

In order to better define the upwelling stations, PP data were obtained from the Ocean Productivity website using the VGPM model following Behrenfeld and Falkowski^94^ (http://www.science.oregonstate.edu/ocean.productivity/index.php).

Picoplankton samples were taken from the initial conditions of the 100% whole seawater (WSW) treatments of grazing experiments (see “Microzooplankton grazing experiments”). PE, Syn and *Proch* were counted by flow cytometry using FACScalibur cytometer (Becton and Dickinson)^95^. Abundance was converted to biomass using the carbon conversion factor of 1500 fgC cell^−1^ for PE^96^, 29 fgC cell^−1^ for *Proch* and 100 fgC cell^−1^ for *Syn*^97^.

### Micro- and mesozooplankton stock measurements

Microplankton samples were collected directly from the Niskin bottle during the daylight cast at 5 m depth (surface), mixed layer (20–30 m) and Chla maximum depth (Table 4). Samples of 500 mL were preserved in alkaline Lugol’s solution until their analysis in the laboratory. An aliquot of 100 mL of each sample was allowed to settle using sedimentation chambers^98^ and analysed on an inverted Olympus IX83 microscope equipped with a motorized focus drive. The microscope was controlled by CellSens software using the automated image acquisition at 200x magnification. More than 25% of total sample area (minimum of 300 organisms counted) was imaged using the functions of Multiple Image Aligning (MIA) and Z-stack. MIA takes pictures of an area and the Z-stack gets images in the Z plane. Identification and counting of organisms was carried out manually from the digital image. Main microplankton groups were identified: Dia, Din, tintinnids and Cil. Din, considered all as µZ, and Cil were counted as <20 μm, 20–40 μm y >40 μm in order to convert abundance to biomass more accurately. The biovolume of each organism was calculated from its equivalent spherical diameter (ESD) and converted to biomass^89,99^.

MZ samples were collected during daylight hours at each station with a Multiple Opening and Closing Net and Environmental Sensing System (MOCNESS) equipped with a 200 μm mesh net at 0–50, 50–100 and 100–200 m depth intervals. Oblique trawls were conducted at a towing speed of ca. 3 knots, measuring the volume of water filtered using a calibrated electronic flowmeter. MZ biomass was directly obtained on board through image processing using the software ZooImage 1, version 1.2-1^100^ and using a conversion factor from Uye^101^.

### Microzooplankton grazing experiments

To estimate µZ grazing upon phytoplankton, dilution experiments were carried out using the 2-treatments method^102^ based on the seawater dilution technique^103,104^. Briefly, seawater in two treatments consisting in 100 and 5% whole seawater (WSW) was incubated for 24 h to obtain the net growth rate of phytoplankton. The 100% WSW treatment is used to measure the net growth rate of phytoplankton (k), while the intrinsic growth rate (μ) is measured from the 5% WSW treatment. µZ grazing rate (g) was obtained from g = μ-k. Negative values of μ were converted to 0.001 d^−1^, while negative values of g were converted to 0 d^−1^ ^28^.

Water for experiments was collected at the surface (5 m depth), mixed layer (20 m) and at the chlorophyll maximum (CM) during the daylight cast (Table 4). Vertical PAR distribution was measured prior to incubation and light profiles were simulated on board incubator using a set of neutral density and blue plastic filters^25^. Temperature was controlled using a series of Titan 2000 coolers. Each experiment was carried out in triplicate using 3.4 L Tedlar® bags during 24 h. The 100% WSW was gently screened with a 200 μm mesh net to avoid MZ, while the filtered seawater was gravity-filtered through 0.2 µm Whatman® Polycap filter. Experiments were run with added nutrients at saturating concentrations in all stations. Nutrient concentration were obtained from Chla concentration observed by Marañón *et al*.^25^ and converted first to C^93^and then to N and P using the Redfield ratio (final nutrient concentrations were: 2–6 μM of NH_4_Cl and 0.1–0.5 μM of Na_2_HPO_4_). Chla and picoplankton were sampled at t = 0 h (initial conditions) and t = 24 h from each treatment (see methods of analysis above).

The impact of µZ grazing on phytoplankton production was estimated using the ratio g:µ for Chla, PE, *Syn* and *Proch*^28^. It should be noted that we added nutrients to the bottles in order to warrant a critical assumption of the dilution method (phytoplankton growth rates should be independent from the dilution level^103^). Thus, we obtained potential growth rates of phytoplankton.

### Diel phytoplankton growth and mortality

In order to study the daily phytoplankton growth and mortality, dilution grazing experiments were carried out using surface waters (5 m depth). Incubations lasted for 24 h, but there was an intermediate sampling at t = 12 h (early in the morning); after 24 h, (near dusk) final samples were taken and the experiments terminated (Table 5). This depth was selected because the signature of the diel rhythm should be stronger at more illuminated layers, and organisms at the surface are less photosensitive than those inhabiting deeper layers. In this sense, natural variations in light such as clouds or waves, as well as manipulation have a lower impact on surface organisms than most light sensitive organisms.

**Table 5:** Location and time sampling during daily phytoplankton growth and mortality experiments at depth of 5 m.

Station	Date	Sampling time	Sampling hour (UTC)
1	05/04/2015	T = 0	16:06
		T = 12	4:05
		T = 24	16:08
2	07/04/2015	T = 0	19:35
		T = 12	8:37
		T = 24	19:33
3	09/04/2015	T = 0	18:39
		T = 12	7:42
		T = 24	18:45
4	11/04/2015	T = 0	19:34
		T = 12	7:40
		T = 24	19:39
5	13/04/2015	T = 0	19:30
		T = 12	07:40
		T = 24	19:35
6	15/04/2019	T = 0	19:30
		T = 12	7:37
		T = 24	19:40
7	17/04/2015	T = 0	19:20
		T = 12	7:25
		T = 24	19:20
8	19/04/2015	T = 0	18:56
		T = 12	7:08
		T = 24	19:00
9	21/04/2015	T = 0	19:35
		T = 12	7:40
		T = 24	19:43
10	23/04/2015	T = 0	21:04
		T = 12	8:55
		T = 24	21:11
11	25/04/2015	T = 0	19:35
		T = 12	7:40
		T = 24	19:46
12	27/04/2015	T = 0	19:37
		T = 12	7:44
		T = 24	19:38

### Statistical analysis

Principal component analysis (PCA) was used to reduce the dimensionality of physical and biological variables (R Project software). We used the cumulative proportion and the histogram of variances by components to determine the total amount of variance explained by the main components, using those were the variance was >60% of. Then, we interpreted each main component in terms of the original variables, examining both graph of influences and, the magnitude and direction of original coefficients. During PCA analysis, no outlier has been eliminated since the cumulative proportion and the proportion of the variance could explain >60% of the variability. Generalized additive modelling (GAM) was used to explore the dependence between biological and physical parameters (R Project software), using factors from PCA (see Supplementary Equations and Supplementary Fig. 1). Kendall Rank correlation coefficients were used to study the relationships between biomass, mortality rates, and environmental variables (Supplementary Table 4). Kendall Rank is preferable to Spearman test because of its robustness and efficiency in the study of populations with scarcely or tied data. For statistical comparisons, a t-test was used for data with a normal distribution and a Wilcoxon-Mann-Whitney for data with no normal distribution. To study the normality of data, a Shapiro-Wilk test was performed. We carried out the Wilcoxon test to investigate differences between growth and mortality during the day and night (Statistica software). The limit between oligotrophic and productive areas was established at 0.5 mgChla m^−3^ in surface and mixed layer^105^.

## Supplementary information

Supplementary material
