# Supplementary material for: Planktonic food web structure and trophic transfer efficiency along a productivity gradient in the tropical and subtropical Atlantic Ocean
Source: Sci Rep. 2019 Feb 14;9:2044. doi: 10.1038/s41598-019-38507-9 (PMC6376012; doi:10.1038/s41598-019-38507-9)
Supplement: Supplementary file 1 — Supplementary material [file 41598_2019_38507_MOESM1_ESM.docx]

**Planktonic food web structure and trophic transfer efficiency along a productivity gradient in the tropical and subtropical Atlantic Ocean**

**Laia Armengol^1*^, Albert Calbet^2^, Gara Franchy^1^, Adriana Rodríguez-Santos^1^, and Santiago Hernández-León^1^**

**^1^**Instituto de Oceanografía y Cambio Global (IOCAG), Universidad de Las Palmas de Gran Canaria, Campus de Taliarte, 35214 Telde, Gran Canaria, Canary Islands, Spain

^2^Institut de Ciències del Mar, CSIC, Passeig Marítim de la Barceloneta 37-49, 08003. Barcelona, Spain.

*Correspondence to Laia Armengol: laia.armengol@ulpgc.es

**SUPPLEMENTARY MATERIAL**

**Supplementary Equations from GAM model for biological variables.**

Chlorophyll *a* (Chla):

$$Chla=43.5236-1.6335 T+0.0007 NO+0.4469 MZ$$

Autotrophic picoeukaryotes (PE):

$$PE=12.96-0.1T-2.93T^{2}-0.07T^{3}-1.49T^{4}-1.12T^{5}-1.55T^{6}-0.47T^{7}-6.05T^{8}-5.71T^{9}-7.32NO+7.09{NO}^{2}- {5.39NO}^{3}+6.01{NO}^{4}-5.54{NO}^{5}+5.76{NO}^{6}+5.73{NO}^{7}+24.58{NO}^{8}+3.29{NO}^{9}+47.82MZ+143.48{MZ}^{2}-8.73{MZ}^{3}-100.37{MZ}^{4}+77.19{MZ}^{5}+3.69{MZ}^{6}-118.46{MZ}^{7}-80{MZ}^{8}-112.74{MZ}^{9}$$

*Synechococcus (Syn):*

$$Syn=4.54+13.19T+32.87T^{2}+9.89T^{3}+17.45T^{4}+10.5T^{5}-2.44T^{6}-0.65T^{7}+33.75T^{8}+22.75T^{9}-\left( 4.36\cdot{10}^{-9} \right)NO+\left( 4.82\cdot{10}^{-9} \right){NO}^{2}- {\left( 2.74\cdot{10}^{-9} \right)NO}^{3}+\left( 2.91\cdot{10}^{-9} \right){NO}^{4}-\left( 2.79\cdot{10}^{-9} \right){NO}^{5}+\left( 2.87\cdot{10}^{-9} \right){NO}^{6}+\left( 2.88\cdot{10}^{-9} \right){NO}^{7}+\left( 1.08\cdot{10}^{-8} \right){NO}^{8}-4.52{NO}^{9}+7.65MZ-22.26{MZ}^{2}+1.53{MZ}^{3}+2.78{MZ}^{4}-13.8{MZ}^{5}-6.37{MZ}^{6}+7.37{MZ}^{7}+28.56{MZ}^{8}-3.39{MZ}^{9}$$

*Prochlorococcus (Prochl):*

$$Prochl=2.02-2.69T-13.86T^{2}-2.71T^{3}-8.29T^{4}-3.73T^{5}-9.91T^{6}-6.74T^{7}-19.38T^{8}+1T^{9}-5.06NO+0.78{NO}^{2}+ {1.11NO}^{3}-1.12{NO}^{4}+\left( 0.83\cdot{10}^{-9} \right){NO}^{5}-\left( 0.89\cdot{10}^{-9} \right){NO}^{6}-\left( 0.91\cdot{10}^{-9} \right){NO}^{7}-\left( 8.21\cdot{10}^{-8} \right){NO}^{8}+4.01{NO}^{9}-\left( 1.29\cdot{10}^{-11} \right)\mu Z-\left( 7.08\cdot{10}^{-12} \right){\mu M}^{2}+\left( 2.58\cdot{10}^{-11} \right){\mu M}^{3}+\left( 5.5\cdot{10}^{-11} \right){\mu M}^{4}-\left( 3.76\cdot{10}^{-11} \right){\mu M}^{5}+\left( 5.9\cdot{10}^{-11} \right){\mu M}^{6}+\left( 5.11\cdot{10}^{-11} \right){\mu M}^{7}+\left( 3.31\cdot{10}^{-10} \right){\mu M}^{8}+0.46{\mu M}^{9}$$

Microzooplankton ($\mu Z$):

$$\mu Z=22.88-\left( 1.09\cdot{10}^{-11} \right)T-\left( 3.46\cdot{10}^{-12} \right)T^{2}+\left( 6.62\cdot{10}^{-13} \right)T^{3}-\left( 1.45\cdot{10}^{-11} \right)T^{4}-\left( 1.33\cdot{10}^{-11} \right)T^{5}-\left( 1.53\cdot{10}^{-11} \right)T^{6}-\left( 3.5\cdot{10}^{-12} \right)T^{7}-\left( 8.4\cdot{10}^{-11} \right)T^{8}-4.33T^{9}+86.3Chla+68.99{Chla}^{2}+ 23.64{Chla}^{3}-47.22{Chla}^{4}+38.08{Chla}^{5}-40.56{Chla}^{6}-40.1{Chla}^{7}+163.23{Chla}^{8}-46.39{Chla}^{9}-8.47MZ+3.24{MZ}^{2}+0.31{MZ}^{3}+1.55{MZ}^{4}-0.92{MZ}^{5}-0.91{MZ}^{6}+1.01{MZ}^{7}+7.01{MZ}^{8}-0.47{MZ}^{9}$$

Mesozooplankton ($\mathrm{MZ}$):

$$MZ=11.33-1.87MZ+3.16{MZ}^{2}+0.58{MZ}^{3}+2.68T^{4}+1.96T^{5}+2.83T^{6}+0.99T^{7}+13.42T^{8}-1.08T^{9}+1.72Chla+7.65{Chla}^{2}-0.08{Chla}^{3}-0.15{Chla}^{4}-0.06{Chla}^{5}+0.2{Chla}^{6}+0.22{Chla}^{7}-8.61{Chla}^{8}+8.14{Chla}^{9}-17.69\mu Z-40.59{\mu M}^{2}-17.68{\mu M}^{3}-36.51{\mu M}^{4}+6.07{\mu M}^{5}-14.63{\mu M}^{6}-10.43{\mu M}^{7}-51.01{\mu M}^{8}-14.05{\mu M}^{9}$$

**Supplementary Figure 1** Correlation between biological factors measured vs. biological factor predicted by GAM for Chlorophyll *a* (Chla), autotrophic picoeukaryotes (PE), *Synechococcus (Syn), Prochlorococcus (Proch),* microzooplankton ($\mu Z$) and mesozooplankton ($\mathrm{MZ}$).


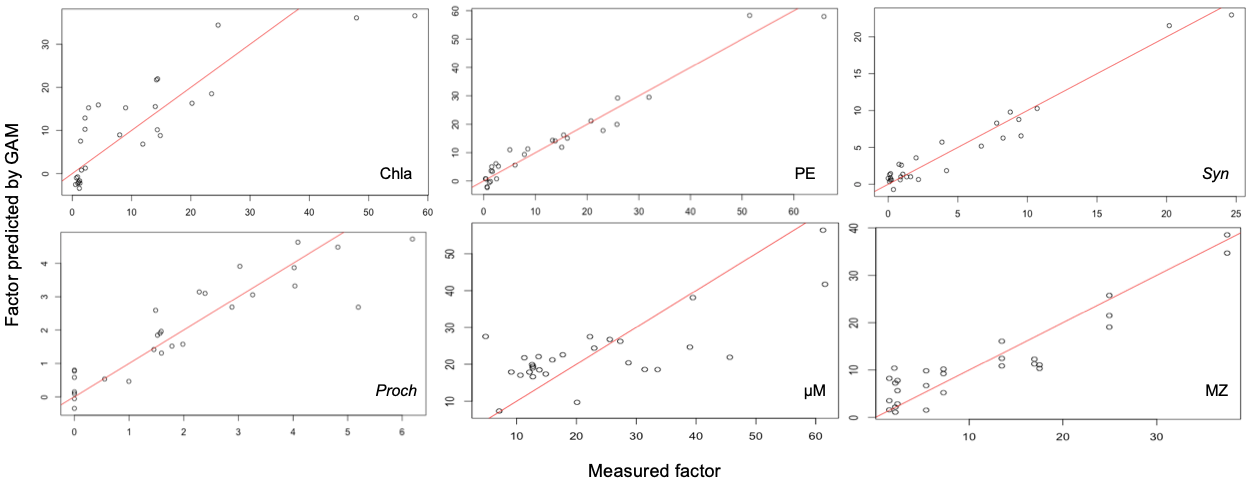


**Supplementary Table 1** Kendall Rank correlations between temperature and biological factor

| **Factor** | **τ** | ***p*-value** |
| --- | --- | --- |
| Chlorophyll *a* | -0.74 | < 0.001 |
| Autotrophic picoeukaryotes | 0.61 | < 0.001 |
| *Synechococcus* | -0.28 | < 0.05 |
| *Prochlorococcus* | 0.40 | < 0.01 |
| Microzooplankton | -0.38 | < 0.01 |
| Dinoflagellates | 0.29 | < 0.05 |
| Naked ciliates | -0.40 | < 0.01 |
| Mesozooplankton | -0.38 | < 0.01 |
|  |  |  |
|  |  |  |
